# A new daily quarter degree sea level anomaly product from CryoSat-2 for ocean science and applications

**DOI:** 10.1038/s41597-023-02300-1

**Published:** 2023-07-21

**Authors:** Christopher J. Banks, Francisco Mir Calafat, Andrew G. P. Shaw, Helen M. Snaith, Christine P. Gommenginger, Jérôme Bouffard

**Affiliations:** 1 National Oceanography Centre, Liverpool, UK; 2 SKYMAT Ltd., Southampton, UK; 3 European Space Agency-ESRIN, Frascati, Italy

**Keywords:** Physical oceanography, Physical oceanography

## Abstract

The European Space Agency launched CryoSat-2 as the first European ice mission in 2010. Its advanced altimeter met primary objectives concerned with sea ice thickness and ice sheets. The value of Cryosat-2 data over global oceans was recognised, and operational products were developed via the CryoSat Ocean Processor (COP). The novel orbit of CryoSat-2 results in a denser coverage of sample points compared to other satellite altimeters. The National Oceanography Centre Sea Level Anomaly (NOCSLA) gridded product is based on interpolating Geophysical Ocean Products (GOP) using weights in space and time. GOP represents the highest quality operational ocean data. NOCSLA is a daily, ¼° sea level anomaly product covering non-coastal oceans between [60°N 60°S] and January 2011 to October 2020. The paper presents the methodology and scientific applications of NOCSLA. Oceanographic features observed are compared against products from other missions, including Rossby waves and El Niño signals. Results show good agreement with other products, confirming the value of Cryosat-2 data for ocean science and applications.

## Background & Summary

The European Space Agency (ESA) CryoSat-2 satellite was launched in April 2010, focussing on measuring the extent and thickness of the Earth’s continental ice sheets and sea ice regions. However, the Synthetic Aperture Interferometric Radar Altimeter (SIRAL) on-board is also able to provide information (sea level, significant wave height and wind speed) on the global marine environment. A dedicated ocean processor (CryoSat Ocean Processor or COP) has been operational since April 2014 routinely providing a number of data products (Fast Delivery Mode, FDM; Interim Ocean Products, IOP; and Geophysical Ocean Products, GOP). FDM are typically available within three hours of acquisition and IOP within three days. FDM have recently been updated to a new improved version (Near real time Ocean Product or NOP). The highest quality level for operational data, and the only products considered in this study, are GOP. The GOP are typically available about 30 days after acquisition and are based on consolidated orbits. The current operational processing version is Baseline C, which was introduced in late 2017. ESA have undertaken a reprocessing of the data from before then to provide a consistent dataset upon which this new product is based. Unlike other satellite altimeters, CryoSat-2 has a long repeat orbit (369 days with a 30-day sub-cycle), as such it provides a denser number of sample points. The choice of orbit was chosen for the need to provide high number of cross-over points in polar regions and also to cover southern Greenland^1^. Whilst the orbit may not be optimal for oceanographic purposes it has been shown to increase the resolution of multi-mission mesoscale fields in energetic regions^2^.

CryoSat-2 can operate in a variety of modes, either in Low Resolution Mode (LRM; conventional pulse limited altimeter), Synthetic Aperture Radar (SAR; enhanced along-track resolution also known as Delay Doppler Altimetry) or Synthetic Aperture Radar Interferometric mode (SARIn; primarily employed at ice sheet edges where SAR is enhanced by the use of a second antenna)^3^. For oceanographic purposes, LRM is of sufficient resolution for most purposes with the notable exception of coastal and polar regions. In coastal regions SAR has proved to be of significant benefit in both lowering noise and measuring closer to the shore^4^ as has specialised retracking of LRM data.

This study is part of a larger ESA funded project concerned with the verification and scientific validation of the CryoSat-2 ocean products, namely CryOcean-QCV. Daily and monthly reports are freely available from https://earth.esa.int/web/sppa/mission-performance/esa-missions/cryosat/quality-control-reports/ocean-product-quality-reports and further details can be found at: https://github.com/ceejbanks/noclsa/blob/main/CryOcean-QCV_technical_note_v1.0.pdf or^1^.

The data presented here provide a regular, daily, ¼° gridded sea level anomaly (SLA) product (NOCSLA; National Oceanography Centre SLA) based on along track SLA data from CryoSat-2 in order to demonstrate the utility of the GOP for undertaking oceanographic studies. The quality of NOCSLA is verified via comparisons with independent SLA data from the Jason-3 satellite, other gridded products and also via a number of case studies.

## Methods

The data for this study cover the period from January 2011 through to October 2020 and are based on GOP data processed using Baseline C. The algorithm described in this section converts the 1 Hz data from Level 2 (L2; along track) data to a spatially and temporally averaged Level 4 (L4) product. This is possible as CryoSat-2 does not have a repeat orbit like other altimeters (e.g., Jason with a 9.92 day repeat orbit) but a non- sun-synchronous 369-day repeat orbit that allows the orbit plane to drift (~0.75°/day). This results in providing far more ground points, whereas it makes looking at temporal changes along track impossible over shorter timescales. A daily, ¼°grid was used as the mid-points for the L4 grid between ± 60°N (referred to as nodes). NOCSLA provides data for the shorter period of 24 January 2011–8 October 2020 (to ensure averaging can occur forwards and backwards in time). For a similar reason, whilst NOCSLA includes data within the range [60°S, 60°N], the L2 data used in the calculations included some poleward of this.

The L2 data were initially filtered to ensure only the inclusion of higher quality data. This involved removing 1 Hz measurements where the quality control flags in the L2 product are invalid or the associated 20 Hz block has less than 10 valid measurements. In addition, data where the absolute SLA was above 3 m were also removed. The algorithm used to calculate the SLA from the L2 data is given in^5^. Briefly, the algorithm involves subtracting the range (corrected for ionospheric and tropospheric path delays as well as sea state bias) from the altitude to obtain the sea surface height (SSH), and then correcting the resulting SSH for barotropic atmospheric effects and the solid Earth, ocean, loading and pole tides. Finally, the mean sea surface is subtracted from the corrected SSH to obtain the SLA. All the corrections are the same as in^5^.

Data selection for each node was based on search radii in both distance (SR_d_) and time (SR_t_) beyond which data were not included in the averaging procedure for that node. In reality, any measurement that satisfies the following was included in the calculation for a given node

1 $$\sqrt{{\left({x}/{{SR}}_{{d}}\right)}^{2}+{\left({t}/{{SR}}_{{t}}\right)}^{2}} < 1$$

where x is the distance in space (km) and t is time in days.

The weighting of each measurement within the spatial and temporal search radii were based on the full width half maximum (FWHM) in both space and time. FWHM is defined as the distance where the weight is equal to half the weighting at the node itself. In space, both SR_d_ and FWHM were taken as a function of the Rossby radius of deformation (RRoD) taken from https://ceoas.oregonstate.edu/rossby_radius (based on^6^). The values of SR and FWHM for both space and time are given in Table 1. Once the weights, in both space and time, have been calculated they were normalised using the maximum weight for a given node and then the weight for each measurement at that node was the product of the two normalised weights. Finally, the combined weight was normalised using the maximum combined weight at each node. Nodes that were either land or where land falls within the spatial search radius were not included.

**Table 1:** Derivation and definition of terms used for calculating weights using full width half maximum (FWHM), search radius (SR), Effective Range.

Variable	FWHM	SR	Effective Range	Weight
Definition	—	$\frac{3}{2}FWHM$	$ef=\frac{FWHM/2}{\sqrt{{\rm{\log }}(2)}}$	—
Space (km)	2 x RRoD	3 x RRoD	$e{f}_{d}=\frac{RRoD}{\sqrt{{\rm{\log }}(2)}}$	exp(−(x^2^/ef_d_^2^))
Time (days)	15	23		exp(−(t^2^/ef_t_^2^))

RRoD is Rossby radius of deformation, x denotes distance in space (km) and t is time (days).

The normalised weights combined were then used to calculate values of weighted mean, standard deviation and median for each node. There was little difference between using the weighted mean or the weighted median (not shown) and so to avoid potential issues with extreme values the weighted median has been taken as the value of NOCSLA throughout the study.

In order to reduce spurious data, a number of additional quality control steps were taken. Firstly, on any given day at a node where the number of observations was less than 10 then the data were removed. Secondly, nodes where the weighted standard deviation was greater than 0.25 m were removed. These choices result in the removal of ~2% of the daily data. This process is repeated, over all days and results in NOCSLA between 24 January 2011 and 8 October 2020.

The data product is based on CryoSat-2 in both LRM and non-LRM (i.e., mostly SAR with some SARIn). There is a small known bias in the SAR mode for Baseline C (~1.5 cm), therefore users can filter LRM/non-LRM data using mask_version and mode_mask. The location of the LRM nodes varies as the geographical mode mask changes over time and the appropriate mode mask version is provided.

## Data Records

The NOCSLA product of sea level anomaly is available in NetCDF format, via figshare^7^, and the variables are described in Table 2. The data in Table 2 are in part taken from the metadata in the file and represent daily, quarter-degree gridded SLA from 24 January 2011–8 October 2020.

**Table 2:** Details of variables provided in NOCSLA.

Variable Name	Long Name	Description
lon	Longitude	Longitude of southwest corner of ¼° cell (180°W–179.5°E)
lat	Latitude	Latitude of southwest corner of 1/4° cell (60°S–59.75°N)
time	Date	Time in days since 1 January 2000.
crs		Required to be CF compliant
sla	Sea level anomaly	Sea level anomaly relative to DTU10^20–22^ (in metres)
land_mask	Land and coastal mask	Fixed mask for land or land within the search radius
mask_version	Mode mark version	Version of lrm_mask used for each time step of sea level anomaly grid as given in Table 3

## Technical Validation

Direct, independent, global validation of the product is difficult as global SLA products that cover the full period of NOCSLA include CryoSat-2 data. A two-stage validation procedure is reported here, firstly as collocations with data from Jason-3 (12^th^ February 2016 onwards) and from the European Space Agency Climate Change Initiative Sea Level version 2^8^ (CCI; up to December 2015). CCI is an ESA lead program that represents the climate system in the form of Essential Climate Variables (ECVs), one of these is sea level. Here we use the global sea level ECV product, which is a monthly, multi-satellite (including CryoSat-2) gridded product with a spatial resolution of 1/4° that covers 1993–2015 (available from 10.5270/esa-sea_level_cci-1993_2015-v_2.0-201612). Altimetry data from the gridded sea surface height product (based on a stable two-satellite constellation) (SEALEVEL_GLO_PHY_CLIMATE_L4_MY_008_057) produced and distributed by the Copernicus Climate Change Service (C3S) are also utilised. These are available at http://marine.copernicus.eu/ and are as daily fields on a ¼° near global grid. For this study, C3S refers to the period from January 2011 to October 2020.

The second part is via oceanographic case studies to demonstrate the agreement of the results using NOCSLA compared to other data sources and/or models.

### Comparison with Jason-3 and Sea Level CCI

The Jason-3 dataset is produced from Sensor Geophysical Data Records (SGDR) provided by the NOAA NODC at ftp://anonymous@ftp.nodc.noaa.gov/pub/data.nodc/jason3/gdr/s_gdr. SGDR are comparable to the GOP with high precision orbits. The Jason-3 dataset was the daily, mean SLA from all the data within each ¼° grid cell (without any weightings). For consistency, the mean difference (of all days and all matching grid cells) between NOCSLA and gridded Jason-3 was subtracted from the Jason-3 values so that the overall mean was the same as for NOCSLA. This offset was 26.90 mm.

For comparison with the CCI, NOCSLA was averaged (median) to provide monthly values of SLA where the location was always LRM. The mean difference (of all months and all matching grid cells) between NOCSLA and CCI was subtracted from the CCI values so that the overall mean was the same as for NOCSLA. This offset was 58.89 mm. Users should note that comparisons with CCI are on a monthly scale and not the native, daily scale of NOCSLA.

**Fig. 1:**
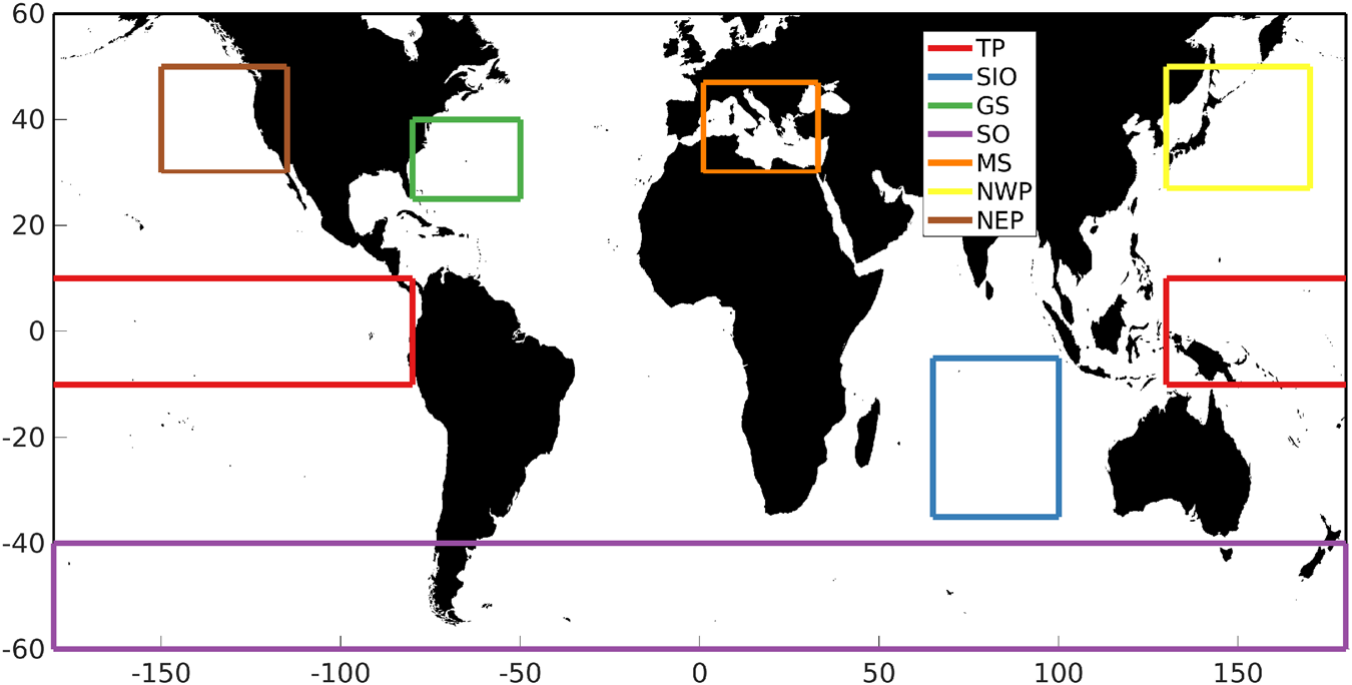
Location of study regions: TP is Tropical Pacific, SIO is Southern Indian Ocean, GS is Gulf Stream, SO is Southern Ocean, MS is Mediterranean Sea, NWP is Northwest Pacific and NEP is Northeast Pacific.

**Fig. 2:**
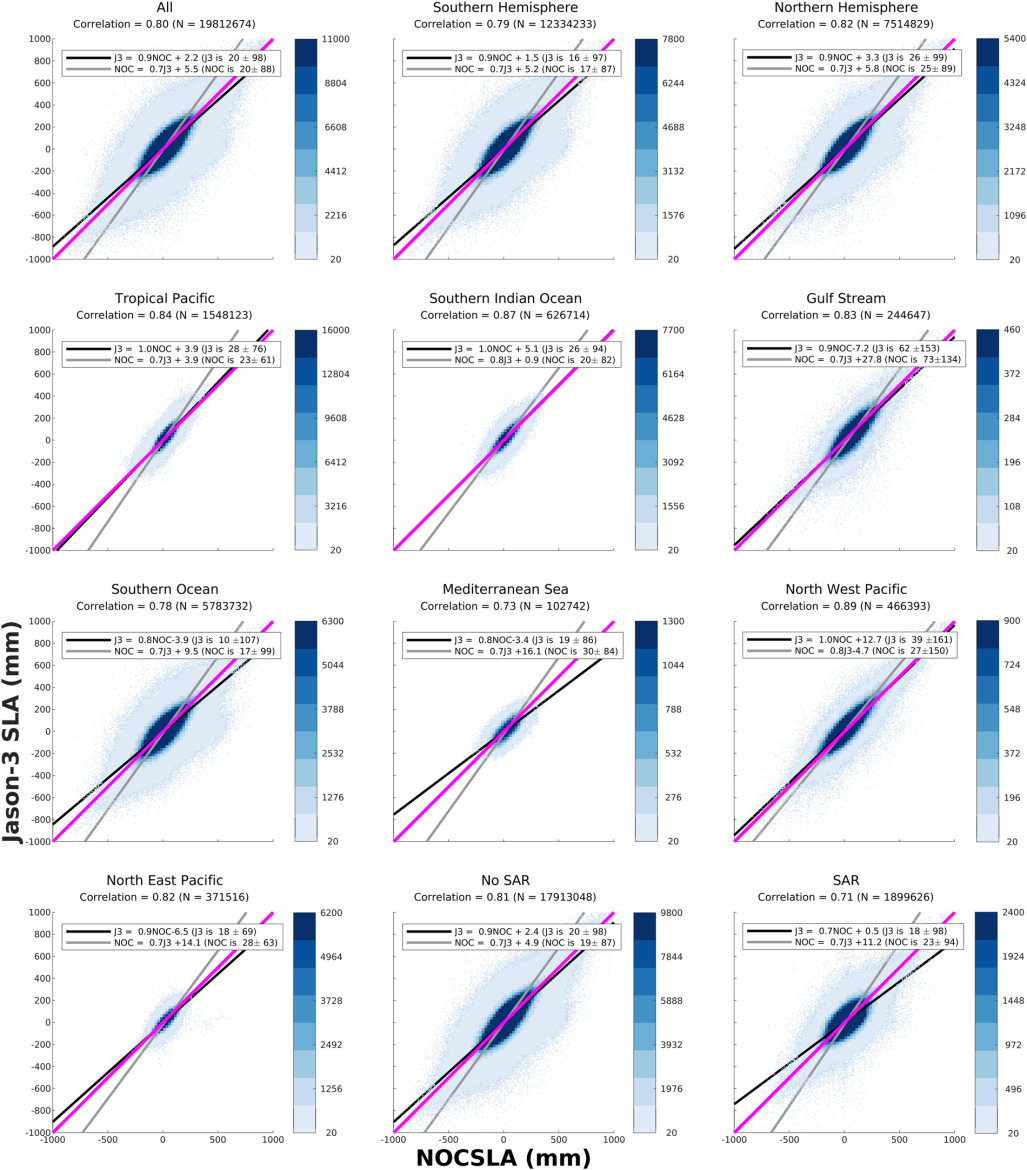
Scattergrams of gridded Jason-3 sea level anomaly (y-axis) with NOCSLA (x-axis) for the various study regions and CryoSat-2 instrument mode (No SAR and SAR). Colour scale is the intensity of the number of points in each 25 mm bin, bins with less than 20 points are located by black dots (note: the colour scale varies by panel). The one-to-one line is the solid magenta line, regression line minimising error in predicting Jason-3 is black line, and regression line minimising error in predicting NOCSLA is grey line. The regression coefficients are given in the legend for each panel and the Pearson correlation coefficient is provided in the title for each panel along with the number of pairs.

**Fig. 3:**
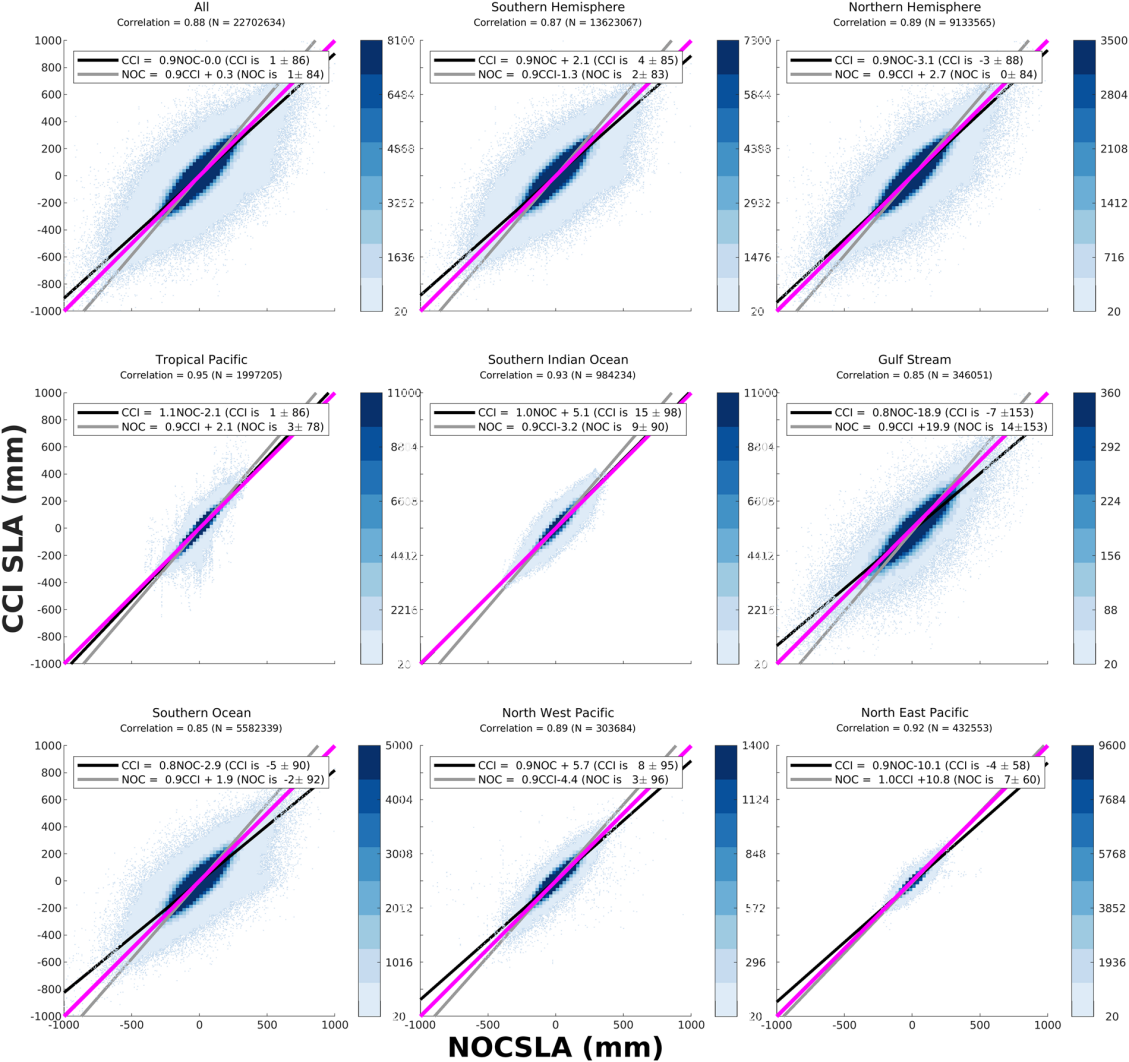
Scattergrams of ESA CCI sea level anomaly (y-axis) with NOCSLA (x-axis) for the various study regions. Colour scale is the intensity of the number of points in each 25 mm bin, bins with less than 20 points are located by black dots (note: the colour scale varies by panel). The one-to-one line is the solid magenta line, regression line minimising error in predicting CCI is black line, and regression line minimising error in predicting NOCSLA is grey line. The regression coefficients are given in the legend for each panel and the Pearson correlation coefficient is provided in the title for each panel along with the number of pairs.

In order to investigate the potential regional variability in agreement a number of study regions were established as shown in Fig. 1 along with considering results by hemisphere. In addition, for Jason-3 the data were split into those specified as LRM and non-LRM according to the geographic mode mask on any given day. Scattergrams of the agreement between NOCSLA and the gridded Jason-3 results are provided in Fig. 2. For the comparison with CCI, scattergrams are presented in Fig. 3. The exclusion of non-LRM NOCSLA data in the CCI comparison, is the reason why there are no results for the Mediterranean Sea in Fig. 3.

For all study regions there is good agreement between gridded Jason-3 and NOCSLA with positive correlations between 0.71 and 0.89 with no signs of systematic bias. The lowest correlation (0.71) is for those regions identified as being non-LRM (i.e., mostly SAR with some SARIn) and an impact of the known SAR bias. For all regions, the highest concentration of match-ups are aligned along the one-to-one lines. As expected, there is more spread in the more dynamic regions (e.g., Gulf Stream, Southern Ocean and Northwest Pacific). For CCI comparison, the lowest correlations are for Southern Ocean and Gulf Stream region, but the correlations are still high at 0.85 for both. In both comparisons, there is some evidence that there is an issue with agreement in the Southern Ocean (also visible to a lesser extent in the All, Southern Hemisphere and LRM panels). Users should take this into account at higher southern latitudes.

### Global and regional sea level comparisons

In order to look at estimates of global and regional sea level a number of other data sources have been utilised. The first dataset is the Sea Level CCI product described in the previous section. The other sources are similar to each other and represent the global mean sea level (GMSL) from the reference missions (i.e. Jason series of satellite altimeters), these are referred to as Beckley^9^, Colorado (https://sealevel.colorado.edu/ with seasonal signals retained) and AVISO (https://www.aviso.altimetry.fr/en/data/products/ocean-indicators-products/mean-sea-level/data-acces.html). Seasonal cycles were included in the downloaded data as these are removed in the methodology described below, so that all data were processed in the same manner as NOCSLA. For AVISO, the data were for the reference missions only, so it provides an independent assessment of GMSL.

**Fig. 4:**
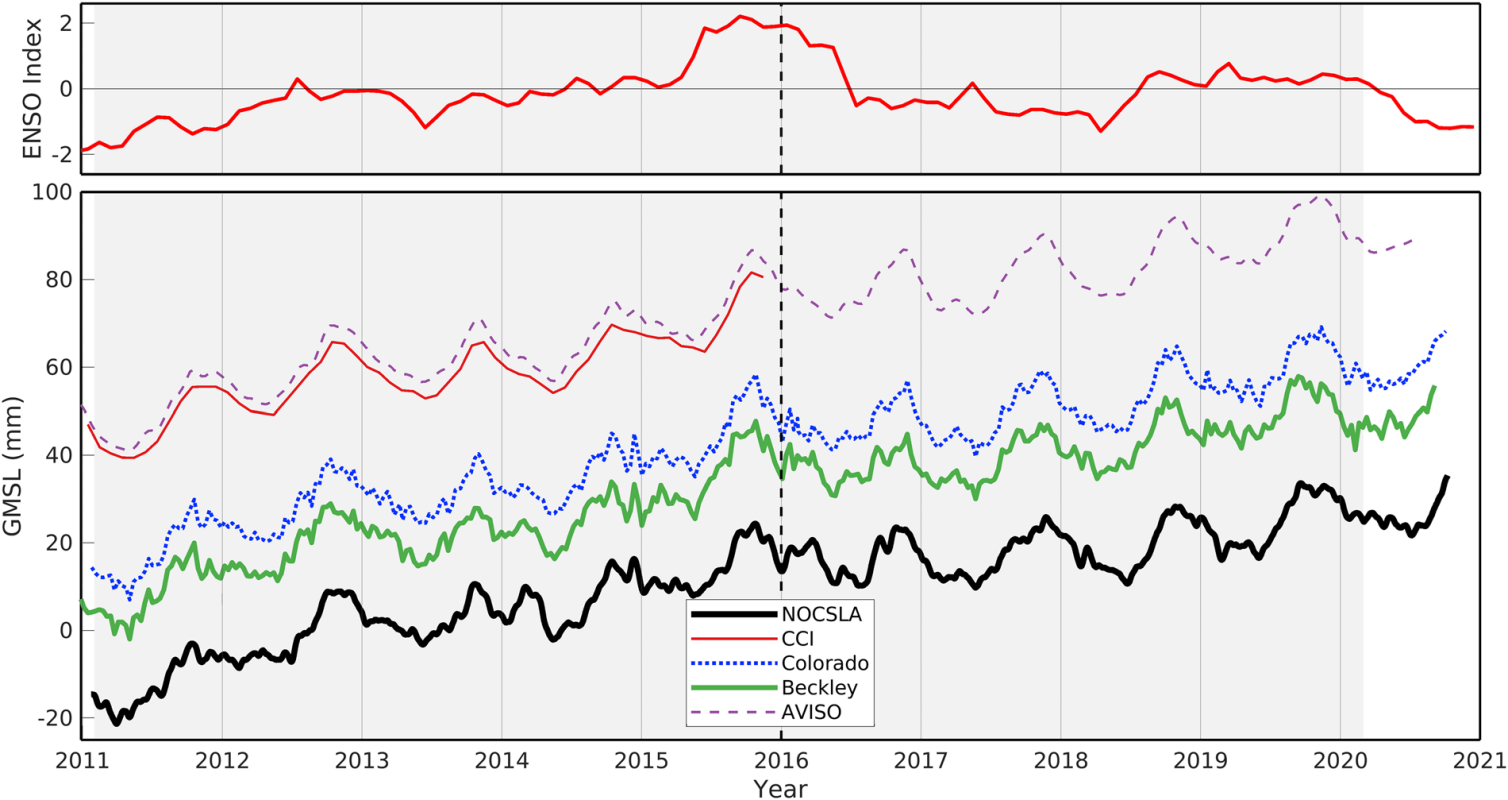
Top panel shows Multivariate ENSO Index (MEI.v2; https://psl.noaa.gov/enso/mei/), a positive value is El Ninõ whereas a negative value is La Niña. Lower panel is Global mean sea level (GMSL) from NOCSLA (solid, thick, black line), ESA Climate Change Initiative version 2 (CCI; thin, solid, red line), Colorado (dotted, blue line), Beckley *et al*.^9^ (thick, solid, green line) and AVISO (thin, dashed, purple line). The shaded period is the full time period presented in Table 4 and the dashed, black line indicates the end of the (2011–2015) CCI period.

The time series of all five GMSL values are provided in Fig. 4. The GMSL from NOCSLA is taken as the daily, mean SLA for all locations and the same split by LRM/non-LRM (the latter is not shown). Only CCI data within [60°N, 60°S] are included to match NOCSLA whereas the other data include all data from the Jason missions (i.e., poleward of ±60°N).

To consider the trends over time, the periods 2011–2015 (full years where CCI data are available), and 2011–2020 (the entire NOCSLA period) are represented in Table 4. Only full years have been used so that the 2011–2020 period covers February 2011 through January 2020. Table 4 provides the annual trends in GMSL from the products alongside the mean of the annual cycle with estimates of the confidence intervals for these study periods.

In order to calculate the trend and amplitudes of the seasonal and semi-seasonal cycles, an ordinary least squared method^10^. is used based on a model estimate of SLA (SLA_model) as given by Eq. 2 below.

2 $${SLA}_{model}=D+Ct+{\sum }_{1}^{n}\left[{B}_{n}{\cos }{\rm{(}}{2}\pi {f}_{n}t{\rm{)}}+{A}_{n}{\sin }{\rm{(}}{2}\pi {f}_{n}t{\rm{)}}\right]$$

where D is a constant, C is the SLA trend (in mm/year), t is time (years), f_n_ is frequency such that f_n_ = n/T_n_ (where T_n_ = 1 year). A and B are both constants. Therefore, the annual cycle is where n is 1 and the semi-annual cycle is where n is 2.

The equation for calculating the amplitude (α) is given in Eq. 3.

3 $${\alpha }_{n}=\sqrt{{A}_{n}^{2}+{B}_{n}^{2}}$$

We account for serial correlation in the residuals from the regression analysis by assuming a regression model with first-order autoregressive errors and estimating the standard error of the regression coefficients using the Prais-Winsten estimator^11^. To estimate the uncertainty in the amplitude (α), we propagate the uncertainty in the regression coefficients A and B through Eq. 3 using standard error propagation formulas under the assumption that such coefficients are independent^12^.

In general, there is good agreement of NOCSLA with the other GMSL including the trends and amplitude of the seasonal cycle. The estimation of trends in GMSL is highly sensitive to the time period used (see for example^13^) and as such estimated trends from the relatively short time series here should be treated with caution in particular those for 2011–2015. For the full time-series analysis, the seasonal cycle amplitude from NOCSLA (5.08 mm) is within the extremes (4.81 mm–6.54 mm) of the other estimates as is the confidence interval. The trend from NOCSLA for this period (4.07 mm/year) is within the confidence intervals of the other time series and the confidence interval is similar to that for Beckley (NOCSLA confidence interval is 0.77 mm/year compared to Beckley 0.71 mm/year).

There is a peak in NOCSLA in early 2014 not seen in CCI or AVISO but observed as a smaller peak in both Colorado and Beckley. The cause of this feature is not clear, although the resulting trough between the resulting double peak (over start of 2014) does coincide with a drop in the ENSO index (top panel of Fig. 4). If no account is made of serial correlation in the trend analysis then the residuals are well correlated with the ENSO index (not shown).

The non-representativeness of the non-LRM is very clear (not shown but has a much higher amplitude in the seasonal cycle compared to the other GMSL values). This is not surprising given the increased areas represented at higher latitudes of non-LRM regions: LRM, in contrast, closely reflects the overall signal.

**Fig. 5:**
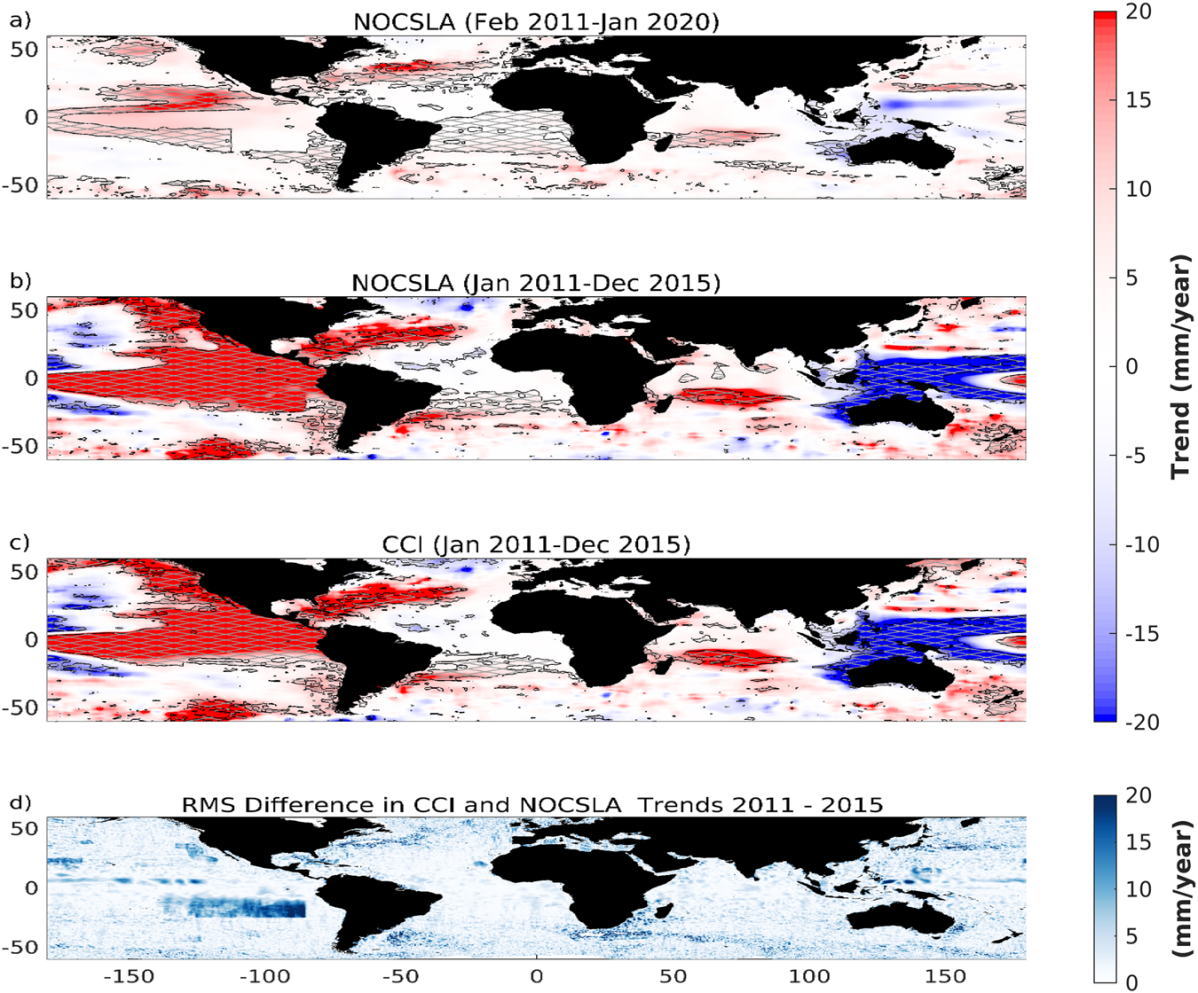
Regional sea level trends from (**a**) NOCSLA over the period February 2011–January 2020, (**b**), NOCSLA over the period January 2011–December 2015, (**c**) ESA Sea Level CCI for same period as (**b**). Colour scale for (**a**–**c**) is trend in mm/year and hatching shows regions where the trend was significantly different from zero (95% confidence). (**d**) shows the root mean square difference between CCI and NOCSLA over the same period as (**b**).

In order to consider NOCSLA on a regional basis the trends in mean sea level (MSL) are calculated for each ¼° over the February 2011 through January 2020 (top panel of Fig. 5). In order to compare with the results from CCI a subset of data were used based on the period January 2011 through December 2015 (middle two panels of Fig. 5). These are based on the methodology described for the global analysis. The trends in Fig. 5 were smoothed in space using a moving window median (5° × 5°) in order to remove small scale features.

The last plot in Fig. 5 shows the RMS difference for the trends obtained from CCI and NOCSLA (2011–2015 period). The SAR box in the tropical south eastern Pacific is clear reflecting the combination of the SAR bias and the changing mode mask, the large SAR box was introduced with mode mask version 3.3 (Table 3). Also clear is highly dynamic regions (higher RMSD) and some locations where there are no trends returned for NOCSLA (Gulf Stream, Kuroshio and Agulhas). The latter are regions where the values of NOCLSA have been filtered.

**Table 3:** Look up table for mask_version.

mask_version	Geographical mode mask version*	Date Period
1	3.1	November 2010 to 17 April 2011
2	3.2	18 April 2011 to 6 May 2012
3	3.3	7 May 2012 to 30 September 2012
4	3.4	1 October 2012 to 6 July 2014
5	3.5	7 July 2014 to 5 October 2014
6	3.6	6 October 2014 to 13 December 2015
7	3.7	14 December 2015 to 6 March 2016
8	3.8	7 March 2016 to 29 January 2017
9	3.9	30 January 2017 to 19 August 2019
10	4.0	19 August 2019 onwards

*see https://earth.esa.int/eogateway/instruments/siral/description.

**Table 4:** Trend (mm/year) and amplitude of annual cycle (α; mm) in Global Mean Sea Level (GMSL) for products detailed in text for inclusive periods 2011–2020 (February 2011 through January 2020; shaded region in Fig. 4) and 2011–2015 (overlap of NOCSLA with CCI).

Source	2011–2020		2011–2015	
	Trend (mm/year)	α (mm)	Trend (mm/year)	α (mm)
NOCSLA	4.07 (0.77)	5.08 (1.01)	6.11 (1.18)	4.52 (1.33)
CCI	—	—	5.32 (1.52)	6.06 (0.90)
Beckley	4.56 (0.71)	4.81 (1.02)	6.02 (1.46)	4.80 (1.37)
Colorado	4.76 (0.55)	5.80 (0.93)	6.05 (1.26)	5.61 (1.26)
AVISO	4.65 (0.56)	6.54 (0.71)	5.59 (1.34)	6.47 (0.93)

The numbers in brackets are the uncertainty at the 95% confidence interval.

The NOCSLA-CCI overlapping time period used here is dominated by the 2015 El Niño event (Fig. 4/Fig. 7) and this is seen in Fig. 5 as the stronger colours in the lower panels compared to top panel. However, the spatial patterns in the middle two panels of Fig. 5 are very similar in terms of both sign and spatial arrangement.

### Comparison with C3S L4

**Fig. 6:**
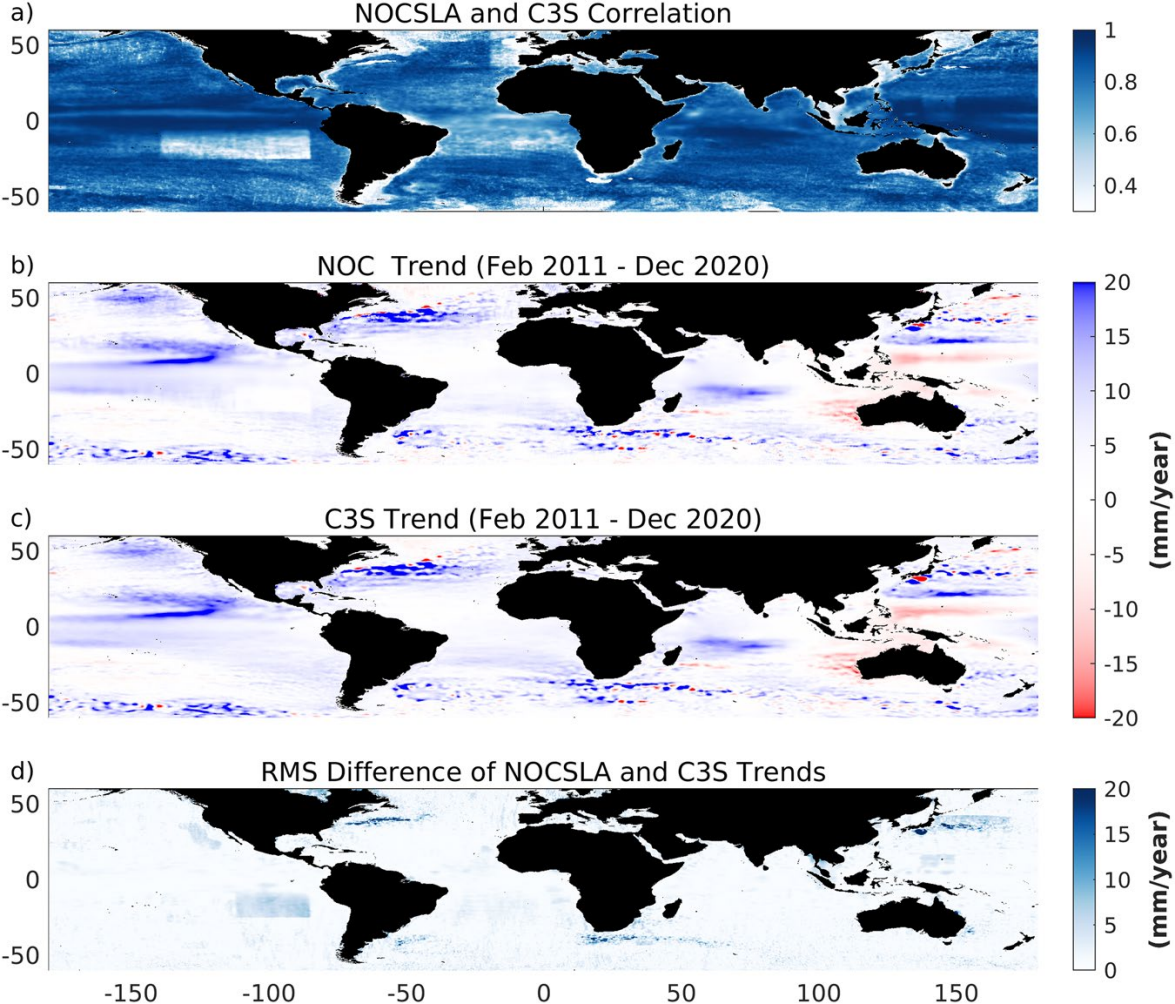
Correlation between NOCSLA and C3S for nonseasonal detrended daily SLA (**a**); trends (mm/year) for the period January 2011 to October 2020 for (**b**) NOCSLA and (**c**) C3S; and (**d**) RMS Difference in NOCSLA and C3S trends.

As an additional validation test, we have also compared NOCSLA with SLA from the C3S product in terms of variability and long-term trends. The comparison is conducted for the period between January 2011 and October 2020. Correlations between the two products for nonseasonal detrended daily values are larger than 0.70 over most (80%) of the global oceans (Fig. 6), with an average value of 0.79. The lowest correlations (<0.7) are mostly found in non-LRM regions, which agrees with the lower correlations with Jason-3 for SAR in Fig. 2.

The trend maps from the two products are almost identical (Fig. 6), showing trends as large as 20 mm/year in regions of high mesoscale activity, particularly along major ocean current such as the Gulf Stream and the Kuroshio. These results give us additional confidence in the quality and robustness of the NOCSLA product.

The lower plot of Fig. 6 shows a similar structure to the equivalent RMS difference plot in Fig. 5 but with a lower mean (lighter colour). The longer time periods (almost 10 years for Fig. 6 compared to 5 years for Fig. 5) result in more stable estimates of the trends and hence lower RMS differences. However, SAR regions and highly dynamic regions still indicate why the user should be cautious when using SAR regions.

### El Niño and southern oscillation signals

**Fig. 7:**
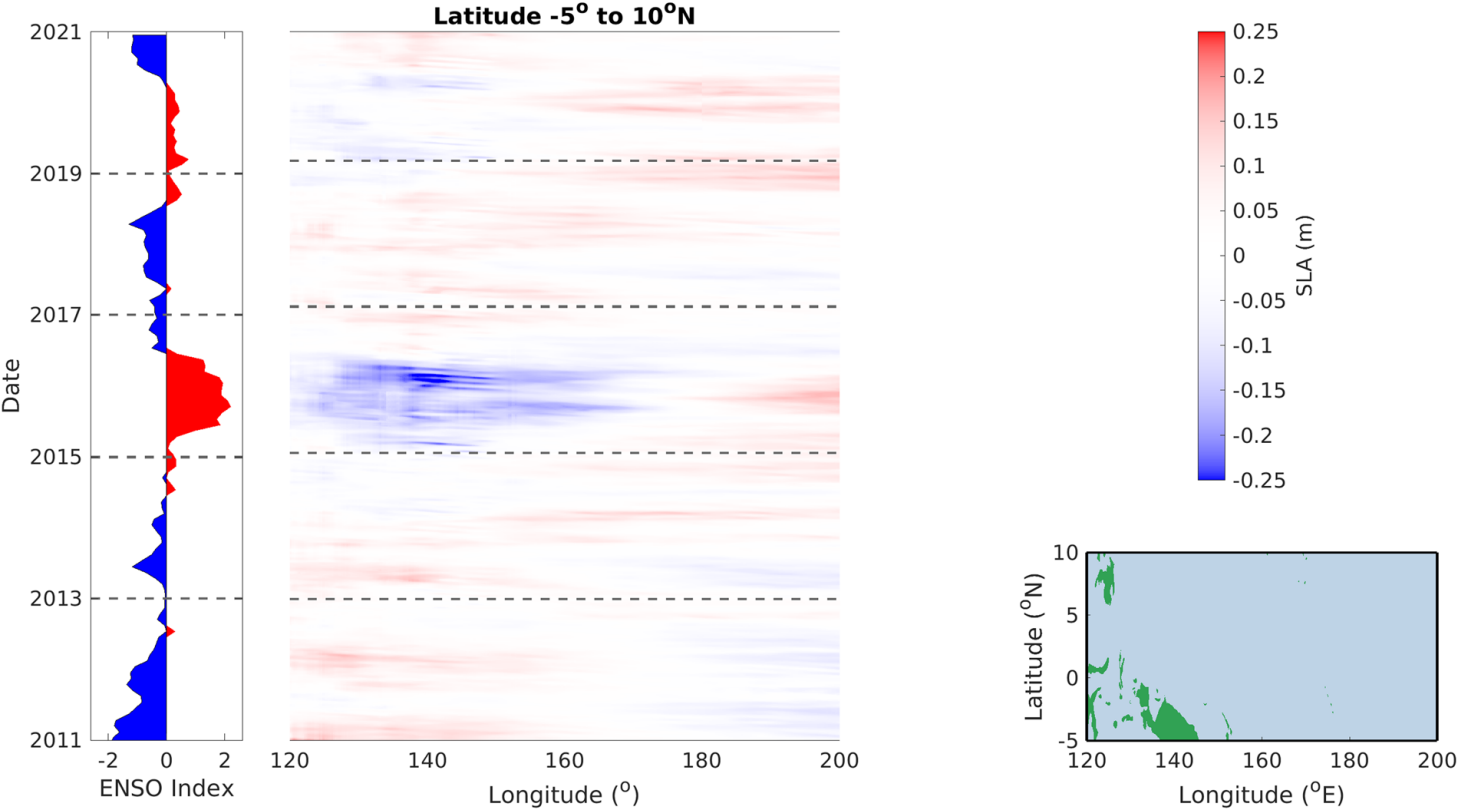
Left hand panel shows Multivariate ENSO Index (MEI.v2; https://psl.noaa.gov/enso/mei/), a positive (red) value is El Ninõ whereas a negative (blue) value is La Niña. The right-hand panel (bottom) shows the area over which this study was conducted and the (top) is the colour scale used in the middle panel. The NOCSLA values in the middle panel are averaged from 5°S to 10°N for each longitude (x-axis) and date (y-axis).

NOCSLA can be used to investigate El Niño and other events in the Tropical Pacific corresponding to the more general El Niño-La Niña-Southern Oscillation (ENSO) as assessed by the Multivariate ENSO Index (MEI.v2; https://psl.noaa.gov/enso/mei/). This is shown in Fig. 7 as latitudinal averaged SLA in a longitude-time plot in the region bounded by [5°S, 10°N] and [120°E, 200°E] as shown in the map in the bottom, right hand corner. The most obvious feature in both NOCSLA and ENSO index is the El Niño event of 2015–2016 corresponding to a large negative anomaly in sea level in the western Pacific and the corresponding positive anomaly in the east. Conversely, a number of La Niña events can be identified in the period 2011–2014, and to a lesser extent about 2017–2018, with negative sea level anomalies in the eastern part of the study region.

As discussed above, the 2015–2016 ENSO has an impact on the results in Fig. 5. Significant anomalies are seen in the Pacific (negative off the coast of South East Asia and positive off the Americas) for the 2011–2015 time period whereas they are not as obvious in the longer timeseries.

### Rossby wave case study

**Fig. 8:**
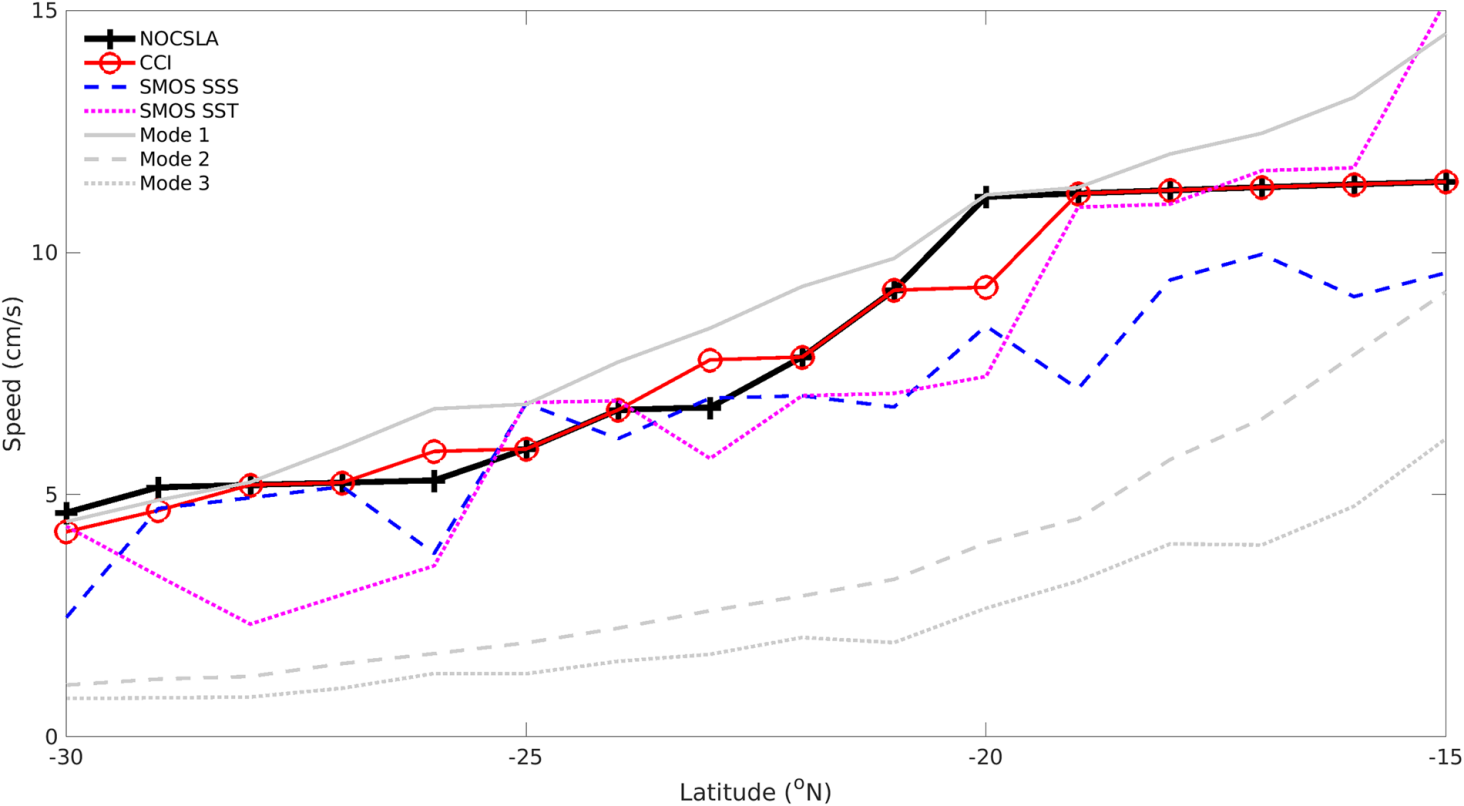
Propagation speeds by latitude for NOCSLA (black cross/solid, black line), CCI (solid, red line/circle). SMOS SSS (blue dashed line) and SMOS SST (dotted, magenta line) are sea surface salinity and sea surface temperature from SMOS ascending passes (see text for further details). 1^st^, 2^nd^ and 3^rd^ baroclinic modes of planetary wave propagation based on^17,18^ (grey dotted, dashed and solid lines respectively^16^).

The final assessment of NOCSLA is based on Rossby (or planetary) waves in the Southern Indian Ocean. Strictly speaking the features identified as Rossby waves in this study could be large non-linear eddies but for brevity are simply referred to as Rossby waves^14,15^. The other data product used in this case study is the ESA CCI product described above. The speeds derived from other variables are taken from^16^, and are the derived Rossby wave speeds for sea surface salinity (SSS) and sea surface temperature (SST) from the ascending passes of the Soil Moisture and Ocean Salinity (SMOS) satellite. The theoretical speed of the first three baroclinic modes of planetary wave propagation based on the extended theory of^17,18^ are also included in Fig. 8, for further details see^16^. NOCSLA data have been averaged to provide a monthly (median) average. A westward propagating filter similar to that described in^19^ was applied to both SLA products. The propagation speeds at different latitudes are then estimated using the Radon Transform on the longitude-time plots over the whole NOCSLA time period between 65°E–105°E.

The speeds calculated from NOCSLA in Fig. 8 are in excellent agreement with those calculated using CCI. It should be remembered that the CCI does not contain any CryoSat-2 data and are therefore independent. The timeframe for CCI is longer than for NOCSLA and the dates are markedly different 1993–2015 compared with 2011–2020 for CCI and NOCSLA respectively.

### Summary

In this paper we have presented a new daily, 1/4° SLA product based only on ESA’s CryoSat-2 satellite. Such a product will enable users to have a single-source dataset with one set of geophysical corrections for comparison with multi-mission products with variable sets of corrections over a relatively long time period. The dataset provides independent, consistent measurements of SLA covering the transition from Jason-2 to Jason-3, and potentially, with an updated product, Sentinel-6 Michael Freilich.

By comparison with other sources of satellite SLA and the various case studies, the validity of NOCSLA in terms of variability and trends has been demonstrated. Depending on the specific requirements of a study (e.g., comparing means from LRM and non-LRM regions) then care must be taken when using non-LRM/SAR data (see Figs. 2, 5, 6). It is hoped that the next reprocessing of CryoSat-2 data (Baseline D) will remove the bias in SAR data and result in improved agreement of LRM and non-LRM data.

In highly dynamic regions (e.g., Gulf Stream), animations of NOCSLA over time (see Supplementary Information) result in non-geophysical signals. These signals are a result of the temporal sampling used at this global scale and work is ongoing to improve the representation of these mesoscale features. Careful choice of local length-scales (in space and time) will improve gridded SLA products using only CryoSat-2 data or, in addition, synergy with other satellite (including other satellite altimeters) and *in situ* data.

## Usage Notes

For the lrm_mask for a given day time(i) then the mask is given by lrm_mask(mask_version(time(i))).

## Supplementary information

NOCSLA animation over time

## Data Availability

The method is fully described in the manuscript, a rudimentary version of the code can be found at https://github.com/ceejbanks/noclsa.
